# Bacterial type III effector protein HopQ inhibits melanoma motility through autophagic degradation of vimentin

**DOI:** 10.1038/s41419-020-2427-y

**Published:** 2020-04-14

**Authors:** Seung-Ho Park, Sung-Jin Yoon, Song Choi, Jun-Seob Kim, Moo-Seung Lee, Seon-Jin Lee, Sang-Hyun Lee, Jeong-Ki Min, Mi-Young Son, Choong-Min Ryu, Jiyun Yoo, Young-Jun Park

**Affiliations:** 1 Environmental Disease Research Center, Korea Research Institute of Bioscience and Biotechnology (KRIBB), Daejeon, Republic of Korea; 2 Infectious Disease Research Center, Korea Research Institute of Bioscience and Biotechnology (KRIBB), Daejeon, Republic of Korea; 3 University of Science and Technology, Daejeon, Republic of Korea; 4 Biotherapeutics Translational Research Center, Korea Research Institute of Bioscience and Biotechnology (KRIBB), Daejeon, Republic of Korea; 5 Stem Cell Convergence Research Center, Korea Research Institute of Bioscience and Biotechnology (KRIBB), Daejeon, Republic of Korea; 6 Division of Applied Life Science (BK21 Plus), Research Institute of Life Sciences, Gyeongsang National University, Jinju, Republic of Korea

**Keywords:** Cancer therapy, Metastasis, Melanoma

## Abstract

Malignant melanoma is a fatal disease that rapidly spreads to the whole body. Treatments have limited efficiency owing to drug resistance and various side effects. *Pseudomonas syringae* pv. *tomato* (*Pto*) is a model bacterial pathogen capable of systemic infection in plants. *Pto* injects the effector protein HopQ into the plant cytosol via a type III secretion machinery and suppresses the host immunity. Intriguingly, host plant proteins regulated by HopQ are conserved even in humans and conferred in tumor metastasis. Nevertheless, the potential for HopQ to regulate human cancer metastasis was unknown. In this study, we addressed the suitability of HopQ as a possible drug against melanoma metastasis. In melanoma cells, overexpressed HopQ is phosphorylated and bound to 14-3-3 through its N-terminal domain, resulting in stronger interaction between HopQ and vimentin. The binding of HopQ to vimentin allowed for degradation of vimentin via p62-dependent selective autophagy. Attenuation of vimentin expression by HopQ inhibited melanoma motility and in vivo metastasis. These findings demonstrated that HopQ directly degraded vimentin in melanoma cells and could be applied to an inhibitor of melanoma metastasis.

## Introduction

Melanoma is a tumor formed by pigment-producing cells called melanocytes, with the highest prevalence among all skin cancers. Depending on the origin of the primary tumor, melanoma can be classified as follows: cutaneous, acral, mucosal, and uveal melanoma. Among these, cutaneous melanoma is the most common type of melanoma and is the leading cause of 75% of skin cancer-related deaths^1^. Cutaneous melanomas are stimulated by aberrant activation of the mitogen-activated protein kinase (MAPK) signaling pathway, owing to BRAF or NRAS mutations, promoting cell proliferation, differentiation, and growth^2^. Furthermore, immunosuppression via surface proteins expressed in melanoma cells, such as programmed cell death protein 1 ligand 1 (PDL1) and PDL2, has a vital role in the development and progression of melanomas^3^. Therefore, RAF or MEK inhibitors, as well as immune checkpoint inhibitors, have been used as drugs for treating melanoma. However, resistance to BRAF-targeted treatment^4^ or immunotherapy^5^ reduces the effectiveness of these treatments. Moreover, BRAF inhibitors can cause serious side effects such as squamous cell carcinoma^6^. Therefore, new therapeutic strategies for melanoma are needed.

Bacterial effector proteins are released into host cells by pathogenic bacteria. In general, this leads to the inhibition of the host immune system or helps pathogens survive^7^. *Pseudomonas syringae* pv. *tomato* (*Pto*) is one of the models used to study the interactions between plants and pathogenic bacteria^8^ and causes diseases in susceptible plants such as tomato and *Arabidopsis thaliana*^9^. In nature, *Pto* injects more than 30 effector proteins, including HopQ into the plant cytosol via a type III secretion machinery and suppresses the host immunity. Once injected into the host, HopQ is phosphorylated by host kinases and binds to the host 14-3-3 protein^10,11^. The 14-3-3 protein is well-conserved among plant as well as animal cells and is known to bind to various signal transduction proteins such as kinases, phosphatases, and transmembrane receptors, thus participating in pathways that are crucial for cancer metastasis^12,13^.

Vimentin is a type III intermediate filament (IF) protein that has a pivotal role in the maintenance of the cytoarchitecture and tissue integrity^14^. Vimentin is also involved in the formation of signaling complexes with cell signaling molecules and other adaptor proteins^15^. It is overexpressed in various types of cancers, including prostate cancer^16^, gastric cancer^17^, breast cancer^18^, lung cancer^19^, and malignant melanoma^20^. In particular, when the epithelial-to-mesenchymal transition (EMT) occurs, vimentin functions as a mesenchymal marker that promotes metastasis of cancer cells^21,22^. In a previous study aimed at identifying biomarkers associated with pulmonary metastasis of melanoma, high vimentin expression was associated with melanoma-derived lung metastasis, and the overexpression of vimentin was frequently observed in primary melanoma patients with hematogenous metastasis^22^. Therefore, regulating the intracellular content of vimentin may be a practical approach to interfere with melanoma metastasis.

Previously, we demonstrated that a type III effector protein HopQ of *Pto* actively interacts with mammalian cellular protein and regulates cell physiology^23^. In this study, we demonstrated that the HopQ from a plant pathogen *Pto* also interacts with 14-3-3 in melanoma cells and regulates vimentin stability, thus inhibiting metastasis of melanoma cells. These data reveal the novel molecular mechanism by which an effector protein of plant pathogenic bacteria inhibits cancer metastasis.

## Materials and methods

### Cell lines

B16F10 (mouse melanoma cell line), SK-MEL-2 (human melanoma cell line), SK-MEL-28 (human melanoma cell line), UACC-257 (human melanoma cell line), and HEK293 (human embryonic kidney cell line) cells were cultured in RPMI (Welgene, Gyeongsan, South Korea) with 10% fetal bovine serum (FBS, RMBIO, Missoula, MT, USA) and 1% antibiotic-antimycotic (Gibco, Grand Island, NY, USA). All cells were maintained at 37 °C with 5% CO_2_ in a humidified chamber. UACC-257 was provided by the Chungnam National University Hospital (Daejeon, South Korea). B16F10, HEK293, SK-MEL-2, and SK-MEL-28 cells were purchased from the Korean Cell Line Bank (KCLB, Seoul, South Korea).

### Antibodies and reagents

Goat anti-Rabbit (111-035-045) and goat anti-Mouse (115-035-062) antibodies were purchased from Jackson ImmunoResearch Laboratories (West Grove, PA, USA). Anti-c-Myc tags (A00704) were purchased from GenScript Corporation (Piscataway, NJ, USA). Anti-pan 14-3-3 (sc-629), anti-14-3-3 beta (sc-628), anti-14-3-3 gamma (sc-731), anti-14-3-3 epsilon (sc-1019), anti-14-3-3 zeta (sc-1019), anti-14-3-3 theta (sc-732), anti-β-actin (sc-47778), anti-GFP (sc-9996), and anti-c-Myc (sc-40) were purchased from Santa Cruz Biotechnology (Santa Cruz, CA, USA). Anti-Vimentin (ab92547) and anti-N-Cadherin (ab12221) were purchased from Abcam (Cambridge, United Kingdom), and anti-LC3B (7543) and anti-p62/SQSTM1 (P0067) were purchased from Sigma-Aldrich (St. Louis, MO, USA). Anti-Ubiquitin (#3933), anti-14-3-3 eta (#9640), anti-14-3-3 tau (#9638), anti-phospho-FOXO1 (#9461), anti-FOXO1 (#2880), anti-p53 (#2524), anti-phospho-AKT (#9271), anti-AKT (#4685), anti-phospho-GSK3β (#9336), anti-GSK3β (#9315), anti-phospho-ERK1/2 (#4370), anti-ERK1/2 (#4695), anti-Snail (#3879), anti-β-Catenin (#8480), anti-Cyclin D1 (#2978), and anti-E-cadherin (#14472) were purchased from Cell Signaling Technology (Danvers, MA, USA). Anti-phospho-serine (05-1000×) was purchased from Millipore (Burlington, MA, USA). Bafilomycin A1 (BafA1) was purchased from Selleckchem (Houston, TX, USA). MG132 was purchased from Calbiochem (San Diego, CA, USA). Mitomycin C and chloroquine (CQ) were purchased from Sigma-Aldrich (St. Louis, MO, USA). Z-VAD-FMK and R18 peptide were purchased from Enzo Life Sciences (Plymouth Meeting, PA, USA).

### Plasmids and transfection

FLAG-tagged HopQ and Myc-tagged HopQ were cloned into the pBICEP vector and the pCMV-Myc-N vector, respectively, using INFUSION HD enzyme (Takara, Mountain View, CA, USA). The Myc-HopQ S51A-expressing vector was constructed using EZchange site-directed mutagenesis kit (Enzynomics, Daejeon, South Korea) following the manufacturer’s instructions for transient expression. In brief, cells were seeded in cell culture plates, incubated for 12 h, and transfected with the indicated plasmids using TransIT-X2 Dynamic Delivery System (Takara Mirus Bio, Madison, WI, USA). After 24 h, the cells were harvested and used for immunoblot analysis.

### RNA interference

RNA interference (RNAi) oligonucleotide 5′-CUGUCUUUGCUGUUACGUU-3′ was used for ATG5, while 5′-CAGACAAGAAGCUCCUUCU-3′ was used for ATG7. A negative control siRNA was implemented as a control (Bioneer, Daejeon, South Korea). In brief, a total of 2 × 10^5^ B16F10 cells were cultured in a 12-well plate for 12 h and transfected with the indicated plasmid, with 50 nM of siRNAs, using a TransIT-X2 Dynamic Delivery System (Takara Mirus Bio, Madison, WI, USA). After 24 h, the cells were harvested and underwent immunoblot analyses.

### Immunoprecipitation and immunoblot analysis

For immunoprecipitation, whole-cell lysates were prepared after transfection, followed by incubation overnight with appropriate antibodies. The magnetic beads (SureBeads; Bio-Rad, Hercules, CA, USA) were washed three times with 0.2% Tween 20 in PBS (PBST). Immunoprecipitated lysates were added to wash beads and incubated for 2 h at 4 °C. The beads were washed three times, and immunoprecipitates were eluted with 2× sample buffer (Bio-Rad, Hercules, CA, USA) and resolved by SDS-PAGE. Proteins were transferred to PVDF membranes (Millipore, Burlington, MA, USA), blocked in 5% skim milk/PBST for 1 h, and further incubated with the indicated antibodies. Immunoblot analysis was carried out using the Clarity Western ECL substrate (Bio-Rad, Hercules, CA, USA) and GelDoc XR + System with Image Lab (Bio-Rad, Hercules, CA, USA).

### Immunofluorescence analysis

B16F10 cells were grown on 12-mm cover glasses in 24-well cell culture plates with complete medium. Cells were then transfected with appropriate plasmids. After 24 h, cells were fixed with 4% paraformaldehyde for 10 min followed by permeabilization with 0.1% Triton X-100 in PBS. After blocking with 1% BSA for 1 h, cells were incubated with the indicated antibodies overnight at 4 °C. After washing three times with PBS, cells were incubated with secondary antibody for 1 h at room temperature and further washed twice with PBS. Cover glasses were mounted with Dako Fluorescent Mounting Medium (Dako, Glostrup, Denmark), and fluorescence was captured under a fluorescence microscope. The DNA dye 4′,6-diamidino-2-phenylindole (DAPI) was used to visualize the nucleus.

### Wound-healing assay and live-cell motility assay

A total of 6 × 10^5^ B16F10 or 1 × 10^6^ SK-MEL-2, SK-MEL-28, and UACC-257 cells were cultured in 6-well plates for 12 h, transfected with indicated plasmids and incubated for an additional 6 h. Cells were pretreated with 40 μg/ml mitomycin C for 2 h. A wound was made in the confluent cell layer by scratching wells in vertical and horizontal directions. Then, cells were washed twice with PBS and incubated with fresh medium. Photographs were taken at the indicated time point using a phase-contrast microscope with a digital camera. For each image, the width of the scratch was measured at ninepoints along the scratch and the cell-covered area was quantified with the ImageJ program. A live-cell motility assay was performed using the ImageXpress Nano Automated Imaging System (Molecular Devices, USA). A total of 5 × 10^3^ B16F10 cells was cultured in 96-well plates for 12 h, transfected with pCMV-Myc-N or Myc-HopQ, and incubated for an additional 12 h. Live-cell motility of ten single B16F10 cells were monitored every 20 min over an 11 h period. Data analysis was performed using MetaXpress® High-Content Image Acquisition and Analysis Software (Molecular Devices, USA).

### Cell migration and invasion assay

Cell migration and invasion potentials were assessed using a Transwell insert (Corning Incorporated, Corning, NY, USA). In total, 2 × 10^5^ of melanoma cells were resuspended in 200 μl of serum-free medium after transfection for 12 h and seeded in the upper compartment of the chamber. After that, 600 μl of complete medium containing 10% FBS was added into the lower chamber. After incubation for 24 h, cells on the membrane in the upper chamber were wiped off with cotton swabs. Cells that migrated to the lower surface were fixed with 4% paraformaldehyde for 10 min and stained with 0.1% Crystal Violet (Sigma, St. Louis, MO, USA) for 20 min. Finally, cells were microscopically imaged. For the invasion assay, the upper surface of the transwell insert membrane was coated with 0.5 mg/ml Matrigel (Corning Incorporated, Corning, NY, USA) and subsequent procedures were carried out similar to the migration assay. The number of migrated and invaded cells were counted in five randomly selected fields.

### Colony formation assay

B16F10 cells were transfected with pCMV-Myc-N or Myc-HopQ for 12 h and trypsinized, and 8 × 10^2^ cells were cultured in a 6-well plate for 7 d. The cells were then fixed with 4% paraformaldehyde and stained with 0.1% Crystal Violet for 20 min. Finally, the cells were washed with distilled water, and the colonies in each well were enumerated with a microscope.

### Real-time PCR

Total RNA was extracted from the cells using NucleoZOL reagent (Macherey-Nagel, Bethlehem, PA, USA) according to the manufacturer’s instructions. For RT-PCR analysis, cDNA was generated with ReverTra Ace qPCR Master Mix with gDNA Remover (TOYOBO, Osaka, Japan). qPCR was performed using specific primers and the ROTOR-Gene Q (QIAGEN, Hilden, Germany) with the THUNDERBIRD SYBR qPCR Mix (TOYOBO, Osaka, Japan). All data were normalized to *RPS18* expression. Primer sequences were as follows: RPS18, forward: 5ʹ-TTCTGGCCAACGGTCTAGACAAC-3ʹ, reverse: 5ʹ-CCAGTGGTCTTGGTGTGCTGA-3ʹ; vimentin, forward: 5ʹ-AAAGCGTGGCTGCC AAGAA-3ʹ, reverse: 5ʹ-ACCTGTCTCCGGTACTCGTTTGA-3ʹ.

### Cell viability assay

For the cell viability assay, pCMV-Myc-N or Myc-HopQ-transfected B16F10 cells were placed in a 96-well plate at a concentration of 3 × 10^3^ cells per well. Each group prepared six individual. Then, cells were incubated for 0, 24, and 48 h before the addition of 10 μl of CCK8 (Dojindo, Kumamoto, Japan) to each well, and incubation for 2 h, followed by absorbance measurement at a wavelength of 450 nm.

### In vivo metastasis

Eight-week-old male C57BL/6 mice used for in vivo metastasis assay and maintained in accordance with the guidelines of the Institutional Review Committee for Animal Care and Use, KRIBB. Five mice/group were injected with B16F10 cells expressing pCMV-Myc-N or Myc-HopQ via the tail vein (3 × 10^5^ cells in 200 μl PBS). After 2 weeks, the mice were sacrificed. The number of lung metastasis was counted.

### Statistical analysis

Sample size was chosen based on the need for statistical power. All experiments were performed triplicate. Data represent mean ± S.D. Data were processed in Microsoft Office Excel 2016 and GraphPad Prism software version 8.1.1 (Prism, La Jolla, CA). Statistical analysis was performed using a *t*-test. *P* ≤ 0.05 was considered statistically significant.

## Results

### HopQ regulates melanoma cell motility

**Fig. 1:**
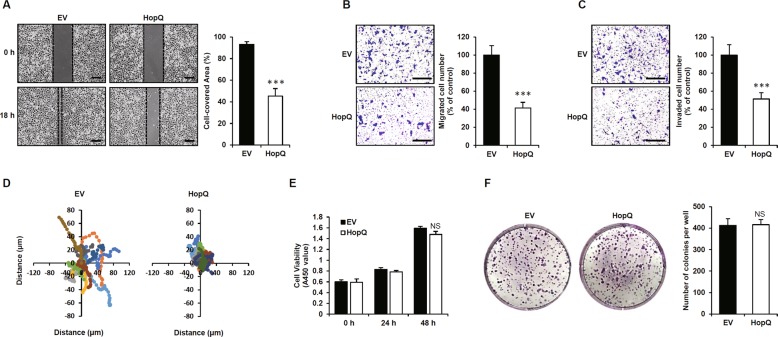
HopQ regulates melanoma cell motility. **a** B16F10 cells were transiently transfected with pCMV-Myc-N (empty vector, EV) or Myc-HopQ for 12 h, then treated with 40 μg/ml mitomycin C for 2 h and scratch wound-healing assays were performed. Bar graphs show the percentage of cell-covered area. (***P ≤ 0.001). Scale bar: 250 μm. **b**, **c** EV- or Myc-HopQ-expressing B16F10 were seeded in transwell chambers and incubated for 24 h. The lower chamber containing 10% FBS was used as a chemoattractant. For the cell invasion assay, the membrane was coated with 10 mg/ml of Matrigel. Bar graphs show the percentage of migrating or invading cells (***P ≤ 0.001). Scale bar: 200 μm. **d** Live-cell tracking of the movement of ten single EV and Myc-HopQ B16F10 cells. Live-cell motility of B16F10 was monitored every 20 min over a 11-h period. **e** B16F10 cells were transfected with EV or Myc-HopQ for 12 h, and cell viability was measured via the CCK8 assay at the indicated time. NS: no significant difference. **f** B16F10 cells were transfected with EV or Myc-HopQ for 12 h, re-plated on 6-well plates, and cultured for 7 d. The cells were fixed and stained with 0.1% Crystal Violet. NS no significant difference.

The effector protein of *Pto*, HopQ, has been shown to function in animal cells, where it regulates actin filament dynamics^23^. Thus, we sought to determine the effect of HopQ on melanoma cell motility. To this end, B16F10 cells were transfected with HopQ and subjected to wound-healing assay. Indeed, the migration of HopQ-expressing B16F10 cells was lower than that of control cells (Fig. 1a). Similarly, transwell assays for tumor cell migration and invasion revealed a significant reduction in the number of migratory and invaded HopQ-expressing B16F10 cells in comparison with the control (Fig. 1b, c). In addition, we confirmed whether HopQ reduces the motility of human melanoma cell lines. The data showed that HopQ-expressing human melanoma cells had decreased motility compared with that of control cells (Supplementary Fig. S1A–C), and live-cell motility assay supported the effect of HopQ on cell migration (Fig. 1d). To examine the effect of HopQ on cell viability, a CCK8 assay was performed, showing that the expression of HopQ did not affect cell viability in both mouse and human melanoma cell lines (Fig. 1e and Supplementary Fig. S1D). Furthermore, no significant changes were observed in the colony-forming potential of the HopQ-expressing B16F10 cells (Fig. 1f). These findings indicated that HopQ had a role in the suppression of melanoma motility.

### The interaction of HopQ with 14-3-3 is required for inhibition of melanoma cell motility

**Fig. 2:**
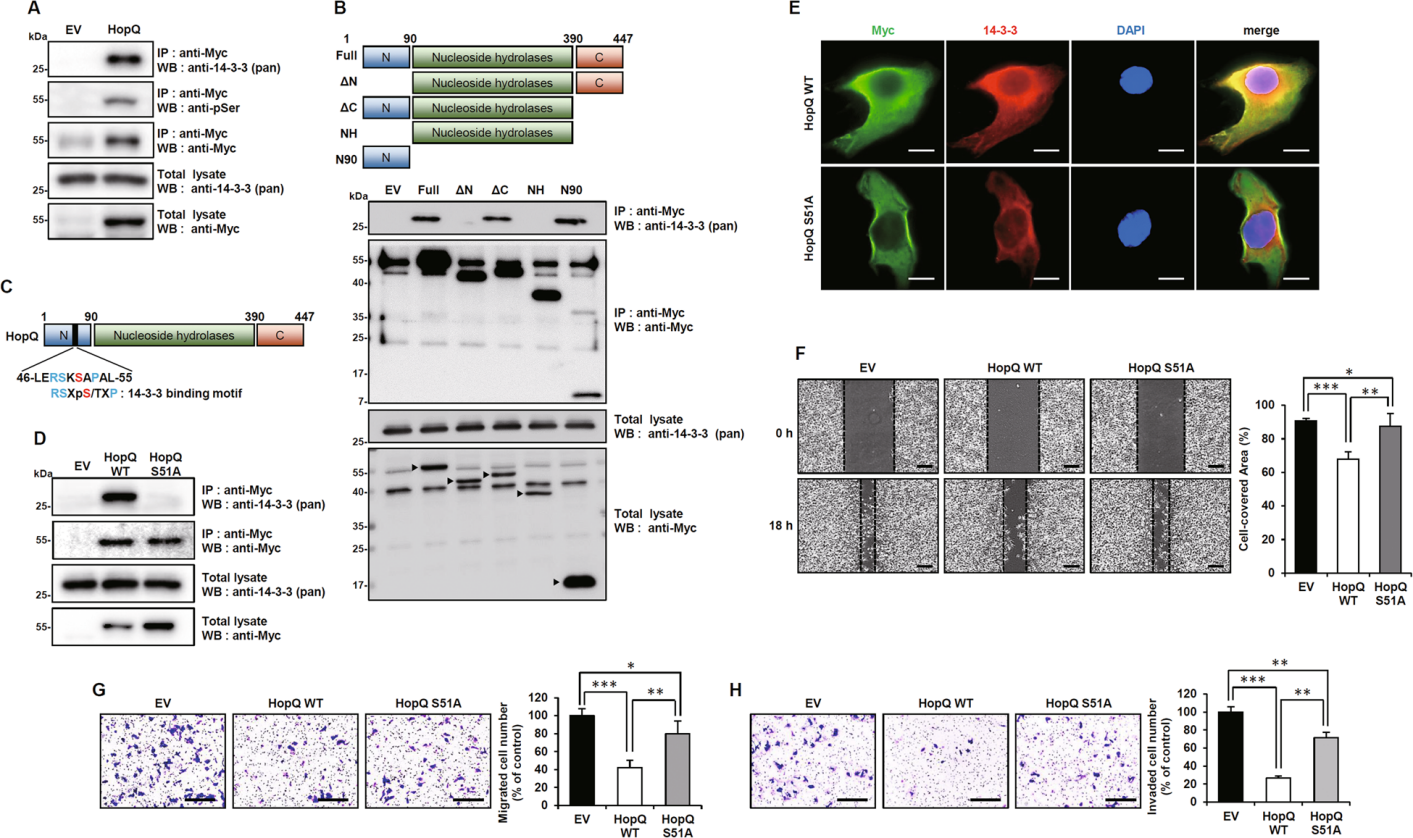
The interaction of HopQ with 14-3-3 is required for inhibition of melanoma cell motility. **a** B16F10 cells were transfected with plasmids expressing EV or Myc-HopQ and whole-cell lysates were immunoprecipitated (IP) with anti-Myc antibody and immunoblot analysis with anti-14-3-3 or anti-pSerine antibodies. **b** B16F10 cells were transfected with EV, Myc-HopQ, or its mutants for 24 h. Whole-cell lysates were then harvested for IP with anti-Myc antibody and immunoblot analysis with the anti-14-3-3 antibody. *Arrowheads* indicate Myc-HopQ mutant bands. **c** Schematic depiction of the 14-3-3-binding motif of HopQ. **d** B16F10 cells were transfected with EV, Myc-HopQ WT, or Myc-HopQ S51A for 24 h. Whole-cell lysates were then harvested for IP with anti-Myc antibody and immunoblot analysis with anti-14-3-3. **e** B16F10 cells were transfected with Myc-HopQ WT or Myc-HopQ S51A for 24 h; cells were then fixed with 4% paraformaldehyde and subjected to confocal microscopy. Scale bar: 20 μm. **f** B16F10 cells transiently expressing EV, Myc-HopQ WT, or Myc-HopQ S51A for 12 h were then treated with 40 μg/ml mitomycin C for 2 h and scratch wound-healing assays were performed. Bar graphs show the percentage of the cell-covered area. (*P ≤ 0.05, **P ≤ 0.01, ***P ≤ 0.001). Scale bar: 250 μm. **g**, **h** EV-, Myc-HopQ-, or Myc-HopQ S51A-expressing B16F10 were seeded in transwell chambers and incubated for 24 h. The lower chamber containing 10% FBS was used as a chemoattractant. For the cell invasion assay, the membrane was coated with 10 mg/ml of Matrigel. Bar graphs show the percentage of migrating or invading cells (*P ≤ 0.05, **P ≤ 0.01, ***P ≤ 0.001). Scale bar: 200 μm.

When HopQ is injected into plant cells, it is phosphorylated and binds to the host 14-3-3 protein to regulate pathogenicity^10,11^. The same events were also observed in HopQ-overexpressing mouse melanoma B16F10 cells (Fig. 2a). Moreover, HopQ bound to almost all 14-3-3 isoforms (Supplementary Fig. S2). To determine which domain of HopQ was responsible for the interaction with 14-3-3, four truncated mutants were generated: ΔN, lacking the N-terminal region; ΔC, lacking the C-terminal region; NH, containing only the nucleoside hydrolase (NH) domain; and N90, containing only an N-terminal 90 amino-acid region. Co-immunoprecipitation experiments with these truncated mutants revealed that the N-terminal domain of HopQ was necessary for binding to 14-3-3 (Fig. 2b). The experiments indicated that HopQ could be phosphorylated in melanoma and bound to 14-3-3. A conserved motif in the HopQ N-terminal domain was found to be involved in 14-3-3 binding (Fig. 2c), and phosphorylation of serine 51 was essential for this binding. To assess whether the effect of HopQ on melanoma migration was due to its interaction with 14-3-3, a mutant in which serine 51 was replaced by an alanine (HopQ S51A) was produced. The HopQ S51A mutant could not interact with 14-3-3 (Fig. 2d). Confocal microscopy further confirmed that HopQ WT co-localized with 14-3-3 in the cytosol, but HopQ S51A did not (Fig. 2e). Further, we examined the effect of HopQ S51A on melanoma wound healing, migration, and invasion. As a result, HopQ S51A did not inhibit wound healing, migration, and invasion in B16F10 cells (Fig. 2f–h). In conclusion, these results suggested that, in melanoma cells, HopQ was phosphorylated and bound to 14-3-3, and that these events were required for HopQ-induced inhibition of B16F10 cell motility.

### HopQ binds to vimentin and inhibits its stability

**Fig. 3:**
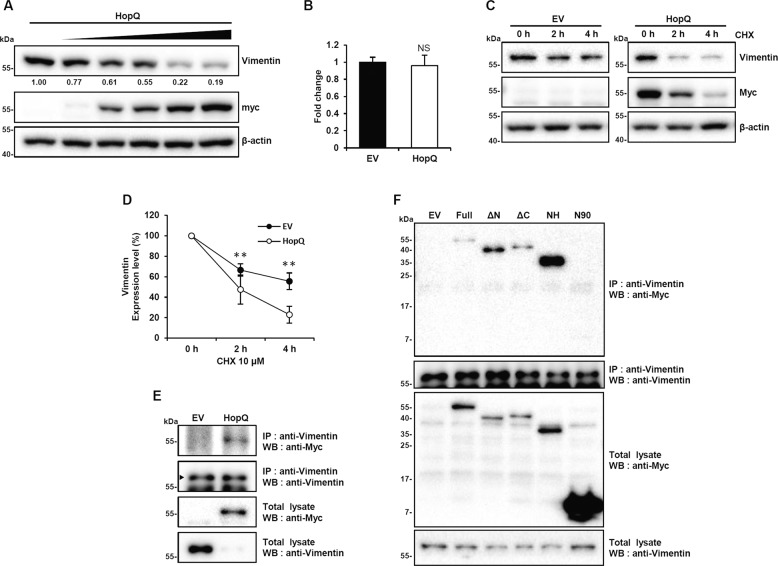
HopQ binds to vimentin and inhibits its stability. **a** B16F10 cells were transfected with increasing doses of Myc-HopQ for 24 h, and cell lysates were collected for immunoblot. **b** EV- or Myc-HopQ-transfected B16F10 cells were analyzed by RT-PCR with vimentin-specific primers. NS no significant difference. **c** B16F10 cells were transfected with Myc-HopQ for 16 h and treated with cycloheximide (CHX) 10 μM at the indicated time points. Cell lysates were then collected for immunoblot. **d** Quantification of the relative levels of vimentin after the treatments described in **c**. (**P ≤ 0.01). The amount of vimentin was normalized to the β-actin level. **e** B16F10 cells were transfected with Myc-HopQ, and whole-cell lysates were used for IP with anti-Vimentin antibody and analyzed by immunoblot with anti-Myc antibody. *Arrowheads* indicate vimentin bands. **f** B16F10 cells were transfected with Myc-HopQ or its mutants for 24 h. Whole-cell lysates were then harvested for IP with anti-Vimentin antibody and immunoblot analysis with anti-Myc antibody.

In an attempt to identify possible signaling pathways regulated by the HopQ/14-3-3 interaction, we examined the expression of 14-3-3 signaling-related proteins involved in cell motility. Interestingly, when HopQ was overexpressed in B16F10 cells, the level of vimentin decreased (Supplementary Fig. S3A). Moreover, vimentin loss increased with the level of HopQ overexpression (Fig. 3a). In addition, we determined whether HopQ decreases vimentin levels in human melanoma cell lines. We found that vimentin levels were significantly reduced in HopQ-expressing human melanoma cell lines (Supplementary Fig. S3B). RT-PCR showed that vimentin mRNA abundance was not altered by HopQ overexpression (Fig. 3b), indicating a post-transcriptional effect. To further investigate whether HopQ induced vimentin degradation, a chase experiment using the protein synthesis inhibitor, cycloheximide (CHX), was performed, confirming that vimentin half-life was significantly reduced upon HopQ overexpression (Fig. 3c, d). Next, immunoprecipitation was performed to determine whether HopQ-induced depletion of vimentin was due to the interaction of HopQ with vimentin. The results revealed that HopQ and endogenous vimentin interacted (Fig. 3e) and that this binding occurred through the NH domain of HopQ (Fig. 3f and Supplementary Fig. S3C). These results indicated that HopQ bound to vimentin, thereby causing vimentin loss.

### The interaction between HopQ and 14-3-3 affects vimentin stability

**Fig. 4:**
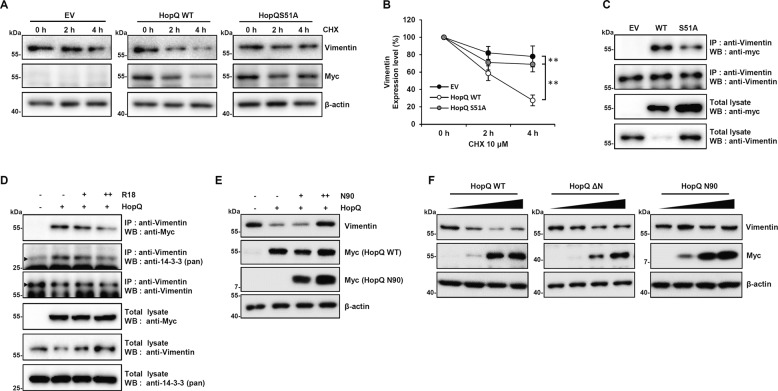
The interaction between HopQ and 14-3-3 affects vimentin stability. **a** B16F10 cells were transfected with Myc-HopQ WT or S51A for 16 h and treated with cycloheximide (CHX) 10 μM at the indicated time points. Cell lysates were then collected for immunoblot. **b** Quantification of the relative levels of vimentin after the treatments described in **a**. (**P ≤ 0.01). The amount of vimentin was normalized to the β-actin level. **c** B16F10 cells were transfected with EV, Myc-HopQ WT, or Myc-HopQ S51A for 24 h. Whole-cell lysates were then harvested for IP with anti-Vimentin antibody and immunoblot analysis with anti-Myc. **d** B16F10 cells were transfected with EV or Myc-HopQ for 12 h and treated with R18 10 μM and 25 μM for an additional 12 h. Whole-cell lysates were then harvested for IP with anti-Vimentin antibody and immunoblot analysis with anti-Myc or anti-14-3-3. *Arrowheads* indicate specific bands. **e** B16F10 cells were transfected with Myc-HopQ with increasing doses of N90 for 24 h, cell lysates were collected for immunoblot. **f** B16F10 cells were transfected with increasing doses of Myc-HopQ WT, ΔN or N90 mutant for 24 h and cell lysates were collected for immunoblot.

To verify whether the effect of HopQ on vimentin expression was dependent on 14-3-3, we examined the impact of HopQ S51A mutant overexpression on the level of vimentin after CHX treatment. Notably, under these conditions, the half-life of vimentin was elevated compared with that of HopQ WT (Fig. 4a, b). In addition, we detected a weaker interaction between HopQ S51A and vimentin than between wild-type HopQ and vimentin (Fig. 4c). Furthermore, treatment with a 14-3-3 inhibitor R18 inhibited the interaction between vimentin with both HopQ and 14-3-3 (Fig. 4d). Moreover, HopQ N90, which binds 14-3-3 but not vimentin (Figs. 2b, 3f), suppressed the vimentin reduction potential of HopQ (Fig. 4e). Thus, these results supported that 14-3-3 was crucial to the regulation of the binding between HopQ and vimentin as well as the decrease in the level of vimentin. Although the NH domain of HopQ was sufficient for binding to vimentin (Fig. 3f), the HopQ S51A mutant, which also contains the NH domain, exhibited decreased affinity for vimentin (Fig. 4c). Therefore, we hypothesized that the HopQ N-terminal domain is important for regulating the affinity of HopQ for vimentin. To verify this hypothesis, we investigated the effect of the N-terminal domain, which is essential for 14-3-3 binding, on vimentin protein stability. Interestingly, the results showed that whereas the HopQ N-terminal deletion mutant (ΔN) caused a slight vimentin depletion to compare with HopQ WT, the N90 mutant, containing only the HopQ N-terminal domain, did not affect the vimentin level (Fig. 4f). These results indicated that the N-terminal domain of HopQ regulates its affinity for vimentin. Overall, our results demonstrated that the effect of HopQ on vimentin expression was dependent on 14-3-3 via regulation of the binding affinity.

### HopQ mediates autophagy-dependent degradation of vimentin

**Fig. 5:**
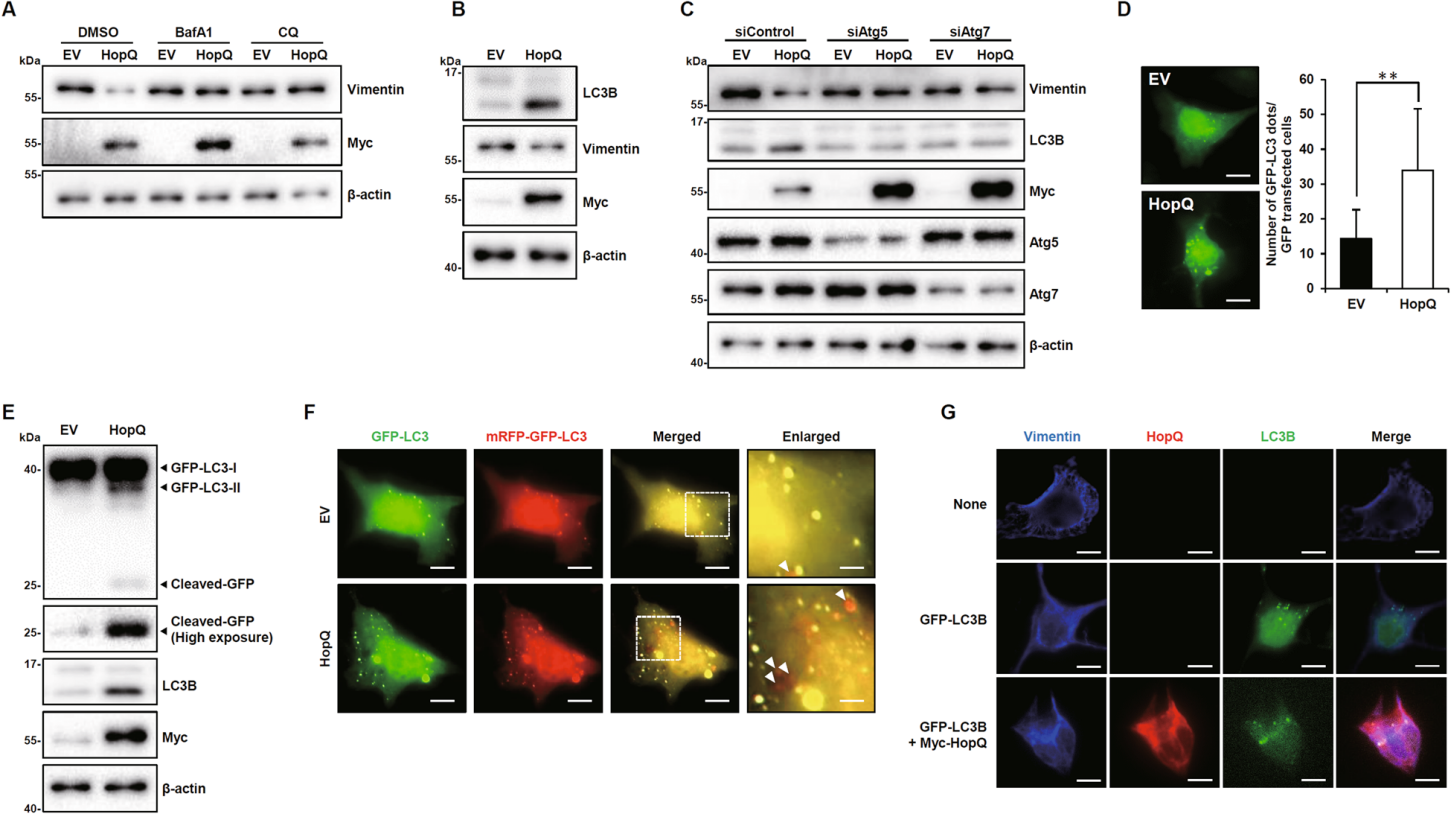
HopQ mediates autophagy-dependent degradation of vimentin. **a** EV or Myc-HopQ-expressing B16F10 cells were treated with bafilomycin A1 (BafA1) 10 nM or chloroquine (CQ) 20 μM for 12 h, then cell lysates were collected for immunoblot. **b** B16F10 cells were transfected with EV or myc-HopQ for 24 h, and cell lysates were collected for immunoblot. **c** B16F10 cells were transfected with EV or Myc-HopQ together with 50 nM of indicated siRNAs for 24 h, and cell lysates were collected for immunoblot. **d** Representative images of green fluorescent protein (GFP)–LC3 punctae in Myc-HopQ-transfected B16F10 cells. The bar graph indicates the number of GFP–LC3 dots per transfected cell. (**P ≤ 0.01). Scale bar: 20 μm. **e** B16F10 cells were transfected with EV or Myc-HopQ, and GFP–LC3 for 24 h, and cell lysates were collected for immunoblot. **f** B16F10 cells were transfected with EV or Myc-HopQ together with mRFP-GFP–LC3 for 24 h, and cells were fixed with 4% paraformaldehyde and subjected to confocal microscopy. In merged GFP and RFP images, yellow dots are visible in autophagosomes, whereas only RFP fluorescence, indicated by red punctae, is observed in autolysosomes (*arrowhead*). Scale bar: 20 μm. Enlarged scale bar: 5 μm. **g** B16F10 cells were either left untransfected or transfected with GFP–LC3B of both Myc-HopQ and GFP–LC3 for 24 h, fixed with 4% paraformaldehyde, and subjected to confocal microscopy. Scale bar: 20 μm.

To date, proteasomal degradation by ubiquitination^24^ and caspase-mediated proteolysis^25^ are the only mechanisms known to control the level of vimentin. Therefore, we sought to determine which of these pathways was involved in HopQ-induced vimentin degradation. We found that neither the proteasome inhibitor, MG132, nor the caspase inhibitor, Z-VAD-FMK, was able to restore vimentin’s protein level in the presence of HopQ overexpression (Supplementary Fig. S4). These results indicated that HopQ activated an alternative degradative pathway. Therefore, autophagosome–lysosome degradation, a distinct mechanism for control of protein levels, was examined. To this end, we used the autophagy inhibitor bafilomycin A1 (BafA1) and chloroquine (CQ) and found that HopQ-mediated degradation of vimentin was completely inhibited by the inhibitors (Fig. 5a). Moreover, the overexpression of HopQ was associated with an increase in the conversion of microtubule-associated protein 1A/1B-light chain 3 (LC3)-I to LC3-II (Fig. 5b), a known marker of autophagic activation. To further validate that ectopic expression of HopQ induces autophagy in melanoma cells, leading to suppressed vimentin expression, we disrupted autophagy by silencing ATG5 and ATG7 with siRNA. The results showed that HopQ-induced vimentin’s inhibitory effect is reduced when autophagy is suppressed (Fig. 5c). When a GFP–LC3 fusion protein was expressed, it accumulated in autophagosomes and increased cytoplasmic punctae^26^. Both GFP–LC3 staining and autophagy were enhanced by HopQ overexpression (Fig. 5d), as assessed by the increase in free GFP resulting from autolysosomal degradation (Fig. 5e). Differences in stability between GFP and RFP at acidic conditions of the autolysosomes reduced GFP fluorescence, but RFP fluorescence was detected. The tandem RFP–GFP–LC3 structure allows for differential labeling of autophagosomes and autolysosomes in yellow and red color, respectively. HopQ increased the autophagic flux of B16F10 cells, as shown by the HopQ-induced increase in red punctae (Fig. 5f). Also, we observed HopQ-dependent vimentin co-localization with LC3B (Fig. 5g). Altogether, these results suggested that HopQ induced vimentin degradation through autolysosomes.

### HopQ induces vimentin interaction with p62 for selective autophagic degradation and inhibits melanoma metastasis

**Fig. 6:**
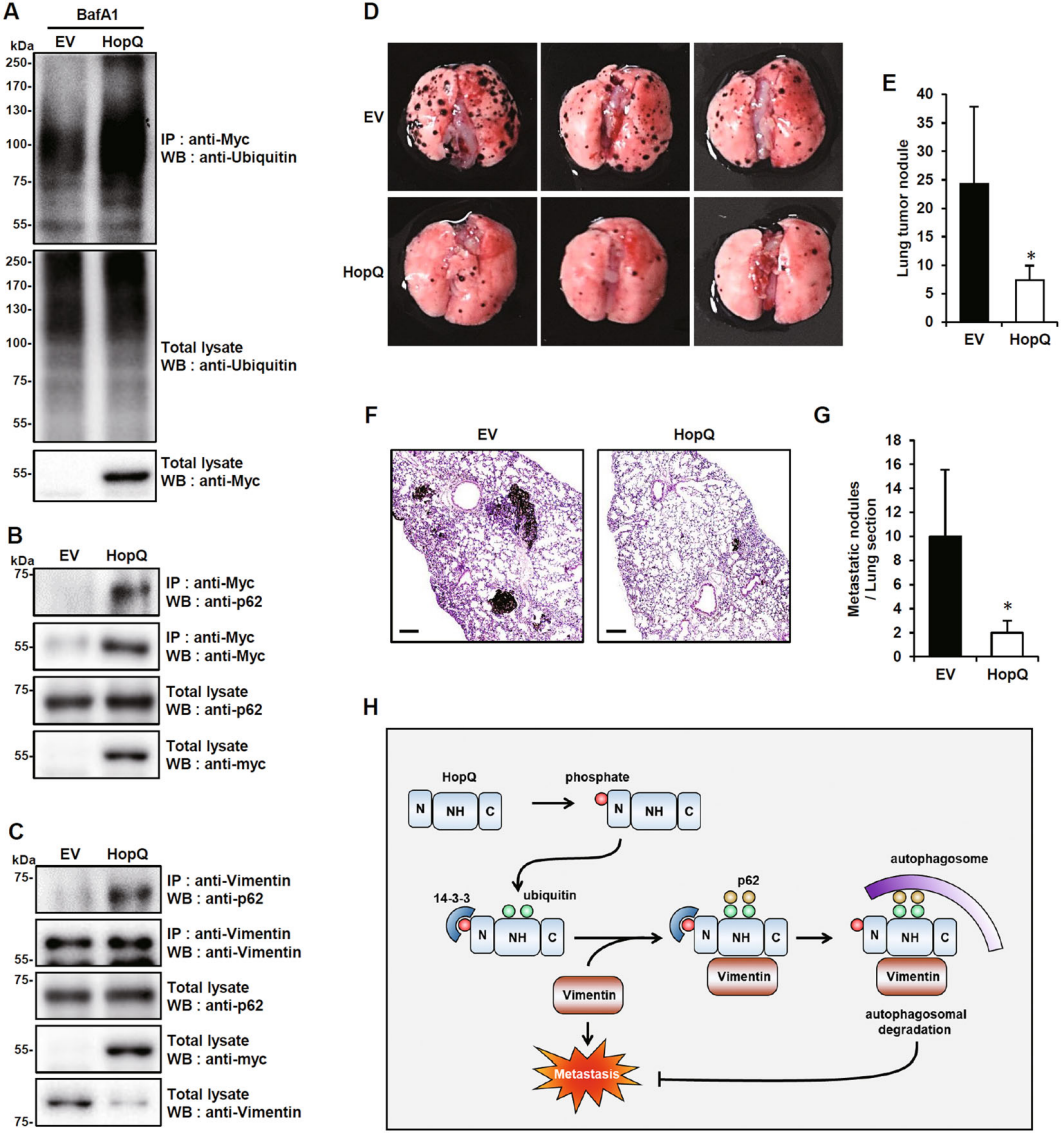
HopQ induces the interaction between vimentin and p62 for selective autophagic degradation and inhibits melanoma metastasis. **a** B16F10 cells were transfected with EV or Myc-HopQ and treated with 10 nM bafilomycin A1 (BafA1) for 12 h, then cell lysates were used for IP with anti-Myc antibody and analyzed by immunoblot with anti-Ubiquitin antibody. **b**, **c** Total lysates of B16F10 cells were transfected with EV or Myc-HopQ, IP with anti-Myc (**b**) or anti-Vimentin (**c**) antibody and analyzed by immunoblot with anti-p62 antibody. **d** EV or Myc-HopQ-expressing B16F10 cells were injected into 8-week-old male C57BL/6 mice (*n* = 5) through the tail vein. The lungs with metastasis were visually counted within 2 weeks after tail vein injection of cells. Representative images of lung metastasis. **e** Quantification of metastatic lung nodules. (*P ≤ 0.05). **f** Representative images of hematoxylin and eosin (H&E)-stained lung tissue sections. Scale bar: 200 μm. **g** Number of lung nodules in H&E-stained lung sections in EV (*N* = 5) and Myc-HopQ (*N* = 5) groups. (*P ≤ 0.05). **h** Proposed model for HopQ-mediated selective autophagic degradation of vimentin in melanoma cells.

Selective autophagic degradation implies the formation of autophagosomes upon recognition of ubiquitinated proteins by cargo receptors^27^. We sought to verify further whether this autophagic mechanism was involved in HopQ-induced vimentin degradation. Indeed, in B16F10 cells, overexpressed HopQ was ubiquitinated (Fig. 6a) and its direct binding to p62, a typical cargo receptor of selective autophagy^28,29^, was confirmed (Fig. 6b). Moreover, HopQ ubiquitination involved the NH domain, and p62 also bound to this domain (Supplementary Fig. S5A, B). Besides, HopQ overexpression increased vimentin binding to p62 (Fig. 6c). This indicated that vimentin underwent HopQ-induced p62-dependent selective autophagic degradation. We, therefore, injected HopQ-overexpressing B16F10 cells into the 8-week-old male C57BL/6 mice through the tail vein to determine whether HopQ overexpression was able to inhibit melanoma metastasis in vivo. As a result, the injection of HopQ-overexpressing B16F10 cells resulted in a decreased number of metastatic tumors in the lungs (Fig. 6d, e). Hematoxylin/eosin (H&E) staining of lung sections confirmed the reduction in metastasis under these conditions (Fig. 6f, g). Altogether, these results indicated that HopQ reduced the in vivo lung metastatic potential of melanoma.

## Discussion

In this study, we determined that HopQ, an effector protein of *P. syringae* pv. *tomato* DC3000, inhibited melanoma metastasis. When HopQ was injected into the host cytosol through a type III secretion system, HopQ bound to the host 14-3-3 protein^10,11^. As 14-3-3 was also known as a regulator of metastasis^12,13^, heterologously expressed HopQ in mammalian cancer cells might be able to modulate metastasis through binding to 14-3-3 protein. Thus, we verified changes in the expression of 14-3-3-interacting molecules, known to be associated with metastasis, under conditions of HopQ overexpression. Surprisingly, the expression of HopQ in melanoma cells caused a previously undescribed intracellular response, i.e., a specific decrease in vimentin protein, apparently unrelated to known mechanisms of 14-3-3-induced metastasis. When HopQ was expressed in melanoma cell, its N-terminal portion bound to 14-3-3, which increased the binding of HopQ to vimentin, whereas no changes in the established 14-3-3 signaling pathways were observed. In B16F10, HopQ underwent increased ubiquitination and subsequent interaction with p62. Under these conditions, p62-dependent selective autophagy occurred, and HopQ-associated vimentin was degraded by autolysosomes (Fig. 6h).

Autophagy has been known as a mechanism allowing for nonspecific mass destruction of unnecessary proteins and cell organelles. Recently, however, many reports have described the degradation of specific proteins by selective autophagy. It is known that proteins such as p62^28,29^, NBR1^30^, and optineurin^31^ recognize ubiquitinated protein aggregates and selectively degrade their targets by binding to autophagosomal LC3. Recent studies have shown that proteins such as the TRIM family members act as secondary receptors and interact with p62 to induce selective autophagosomal degradation of specific targets^29,32^. Moreover, HopM1, a type III effector of *P. syringae* pv. *tomato*, activates autophagy and stimulates the autophagic degradation of proteasomes (proteaphagy)^33^; this shows that the effector proteins and autophagy are closely related. In this study, we have shown that HopQ induces autophagosomal degradation of vimentin as a secondary receptor to interact with p62. It is known that vimentin may interact with p62 in breast cancer cells and that this interaction promotes metastasis^34^. Although it is not known whether this also occurs in melanoma, we reasoned that HopQ could play a similar role in this type of tumor. It is unknown whether HopQ binds to vimentin directly via interaction with p62, which in turn binds to vimentin and forms a complex with ubiquitinated HopQ. However, it seems clear that HopQ-vimentin interaction leads to vimentin degradation through a new p62-dependent mechanism involving selective autophagy.

We also observed that the HopQ-vimentin interaction, and the subsequent degradation of vimentin, depended on 14-3-3 binding to the HopQ N-terminal domain. Notably, although the truncated version of HopQ, lacking the N-terminal domain, did not bind to 14-3-3, it bound to vimentin with higher affinity than the wild-type HopQ did. These results indicated that the N-terminal domain of HopQ prevented the interaction between HopQ and vimentin. However, the binding of 14-3-3 to this domain released this block, allowing for the binding of HopQ to vimentin. As a result, we found that the amount of vimentin was reduced by the HopQ ΔN mutants, which could not bind to 14-3-3. As the NH domain is essential for the binding of HopQ to vimentin, ubiquitination, and binding to p62, it is conceivable that the NH domain alone would exert an inhibitory effect on melanoma metastasis.

In order for HopQ to be developed as a drug for melanoma metastasis, its efficient delivery to melanoma cells is crucial. Recently, studies have demonstrated that YopJ, an effector protein of *Yersinia pestis*, is efficiently delivered to cancer cells by nanoparticles and is more effective than doxorubicin at inducing cancer cell death^35^. In addition to this system, the development of various delivery systems to introduce HopQ to metastatic cancer cells or tumors is considered to be a part of further research. Therapeutic strategies targeting BRAF and MAPK, or based on immune checkpoint inhibitors, still have limited efficacy. Several reports demonstrated that the heterogeneity related to the genetic changes of malignant melanoma^36,37^ or in the gut microbiome^38,39^ of patients might significantly affect the therapeutic responses. Advanced therapies for malignant melanoma are required to overcome the tumor resistance to approved drugs that is due to both the intrinsic heterogeneity of melanoma and the variability of the patient immune system. In conclusion, this work suggests that a bacterial effector protein HopQ, which effectively removes vimentin from melanoma cells, may act as a novel therapeutic target for the treatment of metastatic melanoma.

In conclusion, our study demonstrated that the effector protein HopQ from a plant pathogenic bacterium *Pto* interacts with a human protein 14-3-3 in melanoma cells and decreasing vimentin stability, thus inhibiting metastasis of melanoma cells. HopQ overexpression in the melanoma cell lines reduced its motility and decreased lung metastasis. These effects were due to p62-dependent selective autophagic degradation of vimentin following the interaction between 14-3-3 and HopQ, indicating that HopQ may be useful as an inhibitor of melanoma metastasis.

## Supplementary information

Supplementary Figure legends

Supplementary Figure S1

Supplementary Figure S2

Supplementary Figure S3

Supplementary Figure S4

Supplementary Figure S5
