# Supplementary material for: Bacterial type III effector protein HopQ inhibits melanoma motility through autophagic degradation of vimentin
Source: Cell Death Dis. 2020 Apr 14;11(4):231. doi: 10.1038/s41419-020-2427-y (PMC7156461; doi:10.1038/s41419-020-2427-y)
Supplement: Supplementary file 1 — Supplementary Figure legends [file 41419_2020_2427_MOESM1_ESM.docx]

**Supplymentary Figure 1** HopQ regulates human melanoma cell motility. **a** SK-MEL-2, SK-MEL-28, and UACC-257 cells transiently expressing pCMV-Myc-N (EV) or Myc-HopQ for 12 h were treated with 40 µg/ml mitomycin-C for 2 h and scratch wound healing assays were performed. Bar graphs show the percentage of the cell-covered area. (**P*≤0.05, ***P*≤0.01, ****P*≤0.001). Scale bar: 250 μm. **b-c** EV- or Myc-HopQ-expressing human melanoma cells were seeded in transwell chambers and incubated for 24 h. The lower chamber containing 10% FBS was used as a chemoattractant. For the invasion assay (**c**), the membrane was coated with 10 mg/ml Matrigel. Bar graphs show the percentage of migrating or invading cells. (**P*≤0.05, ***P*≤0.01, ****P*≤0.001). Scale bar: 200 μm. **d** SK-MEL-2, SK-MEL-28, and UACC-257 cells were transfected with EV or Myc-HopQ for 12 h, and cell viability was measured using the CCK8 assay at the indicated time. NS: no significant difference.

**Supplymentary Figure 2** HopQ interacts with all 14-3-3 isoforms. HEK293 cells were transfected with pBICEP or Flag-HopQ and whole-cell lysates were immunoprecipitated with anti-Flag antibody and analyzed via immunoblotting with the indicated antibodies.

**Supplymentary Figure 3** Effects of HopQ protein on 14-3-3 signaling-related proteins and the interaction between vimentin and the NH domain of HopQ. **a** B16F10 cells were transfected with EV or Myc-HopQ for 24 h and cell lysates were harvested for immunoblotting with the indicated antibodies. **b** SK-MEL-2, SK-MEL-28, and UACC-257 cells were transfected with EV or Myc-HopQ for 24 h, and lysates were harvested for immunoblotting with the indicated antibodies. **c** B16F10 cells were transfected with Myc-HopQ or its mutants for 24 h. Whole-cell lysates were then harvested for IP with anti-Myc antibody and immunoblot analysis with anti-Vimentin antibody.

**Supplymentary Figure 4** Vimentin degradation does not occur through proteasome- or caspase-dependent mechanisms. EV or Myc-HopQ expressing B16F10 cells were treated with 10 µM MG132 for 12 h or 10 µM Z-VAD-FMK for 90 min. Thereafter, cell lysates were harvested for immunoblotting.

**Supplymentary Figure 5** The NH domain of HopQ is ubiquitinated and required for its interaction with p62. **a-b** B16F10 cells were transfected with Myc-HopQ or its mutants for 24 h. Whole-cell lysates were harvested for immunoprecipitation with anti-Myc (**a**) or anti-p62 antibodies (**b**) and immunoblot analysis with anti-ubiquitin, anti-p62, or anti-Myc antibodies, as indicated.
