# Supplementary figures and images for: Bacterial type III effector protein HopQ inhibits melanoma motility through autophagic degradation of vimentin
Source: Cell Death Dis. 2020 Apr 14;11(4):231. doi: 10.1038/s41419-020-2427-y (PMC7156461; doi:10.1038/s41419-020-2427-y)

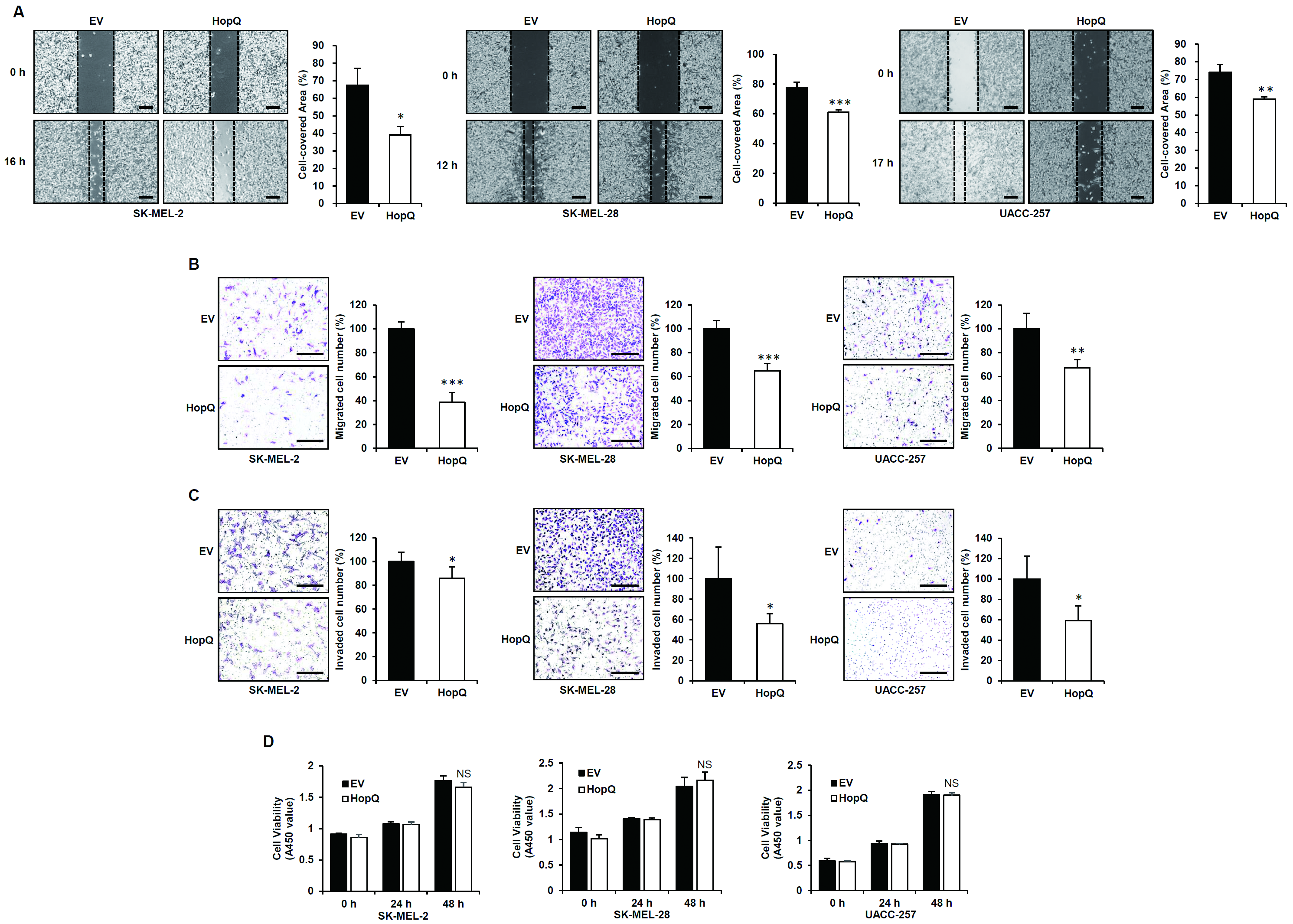

Supplement: Supplementary file 2 — Supplementary Figure S1 [file 41419_2020_2427_MOESM2_ESM.tif]

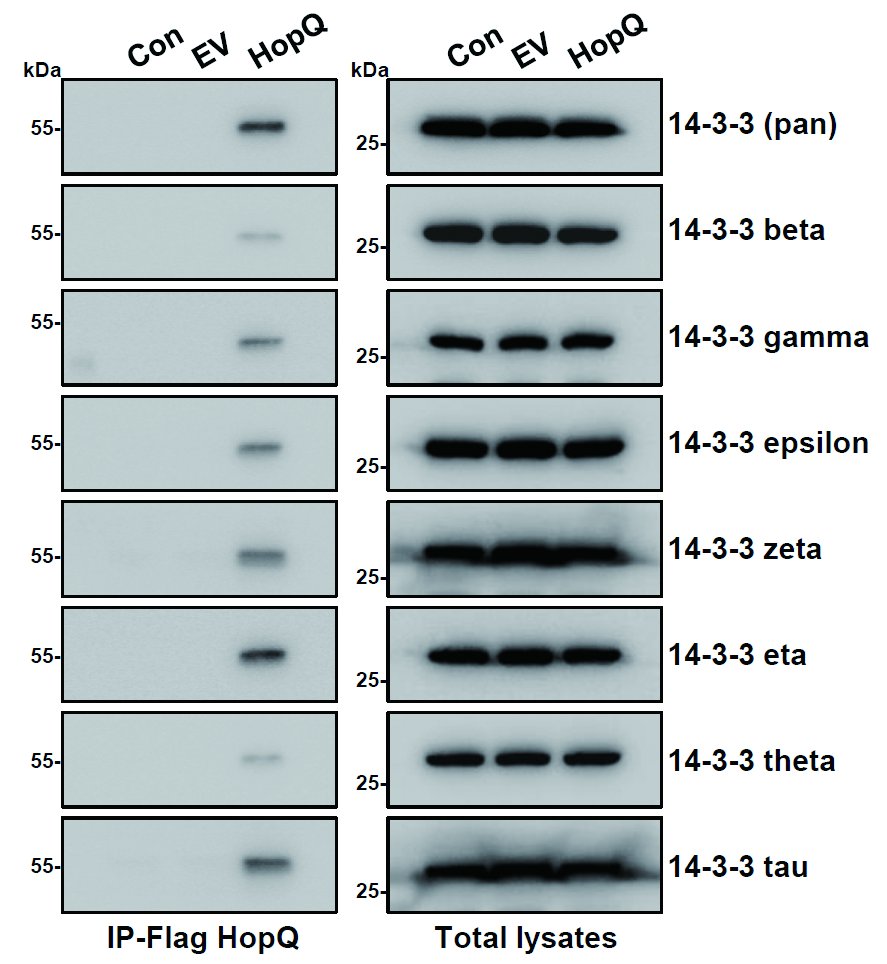

Supplement: Supplementary file 3 — Supplementary Figure S2 [file 41419_2020_2427_MOESM3_ESM.tif]

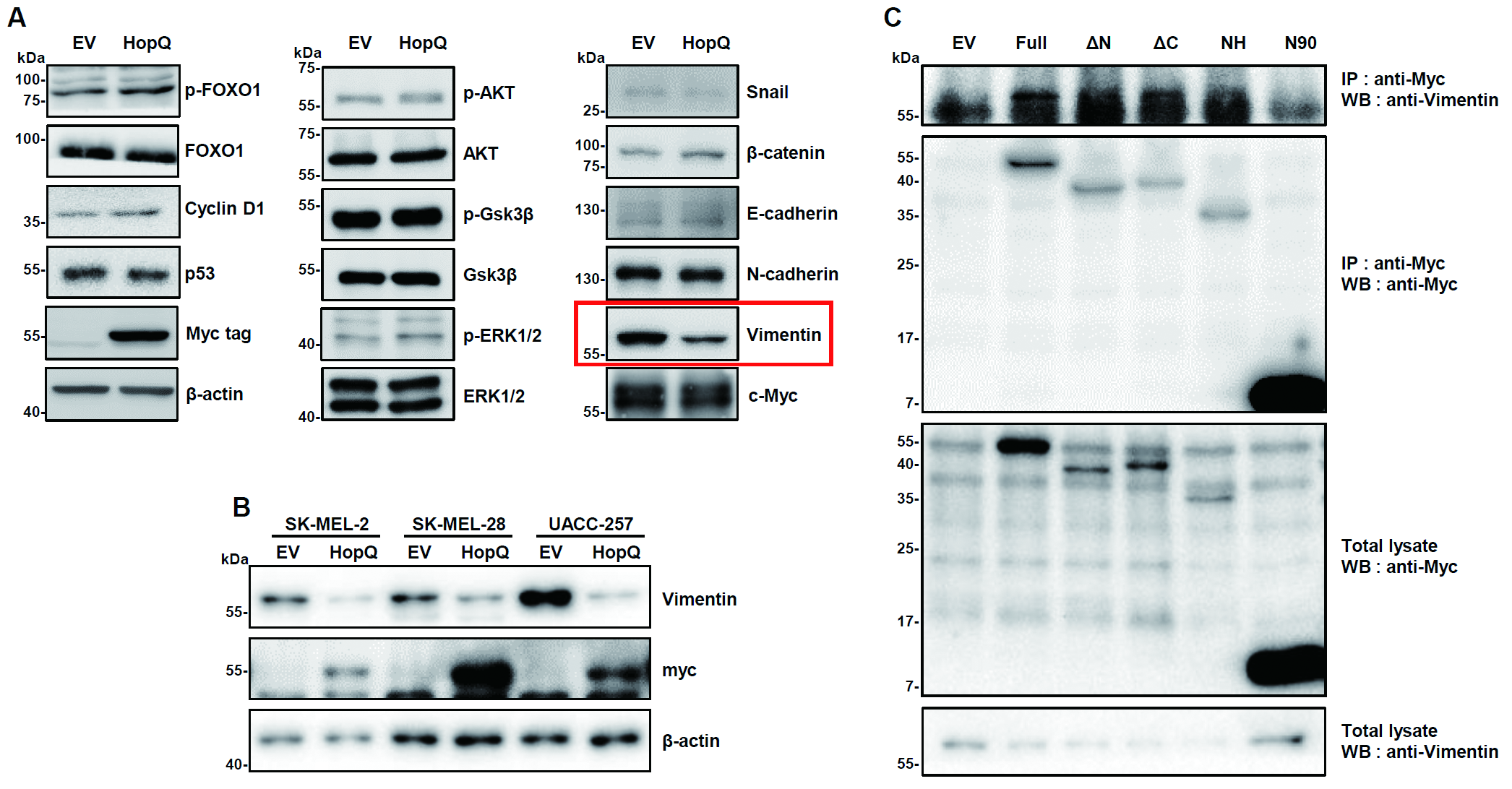

Supplement: Supplementary file 4 — Supplementary Figure S3 [file 41419_2020_2427_MOESM4_ESM.tif]

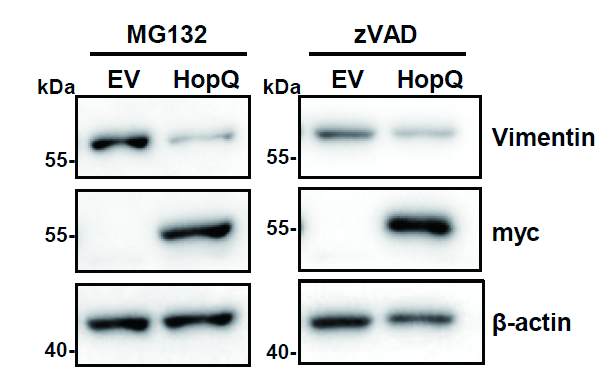

Supplement: Supplementary file 5 — Supplementary Figure S4 [file 41419_2020_2427_MOESM5_ESM.tif]

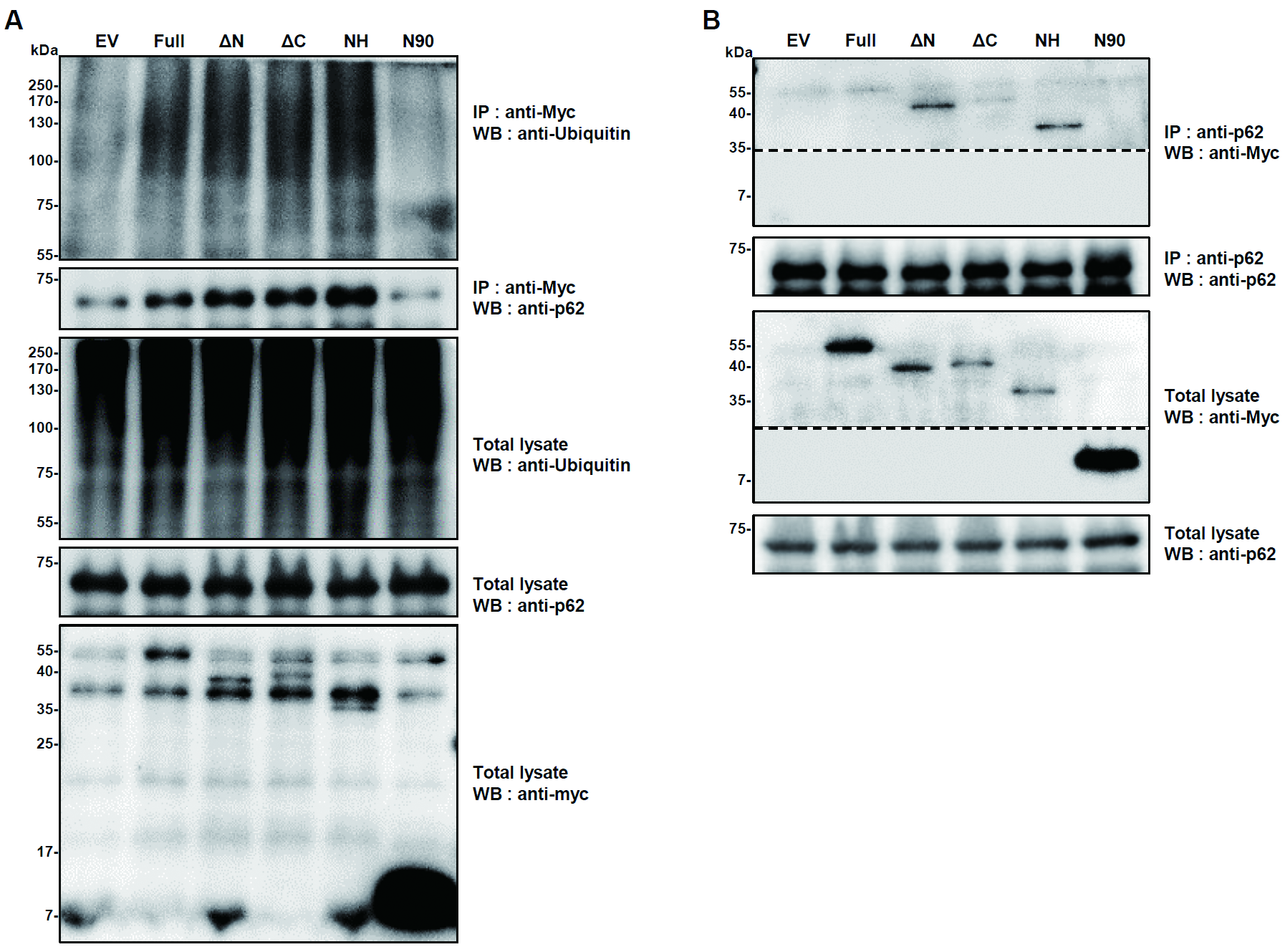

Supplement: Supplementary file 6 — Supplementary Figure S5 [file 41419_2020_2427_MOESM6_ESM.tif]
